# Talking ‘Bout Your Generation: Young Adults’ Beliefs About Older Adults

**DOI:** 10.1093/geroni/igaf122.1594

**Published:** 2025-12-31

**Authors:** Anna Pot

**Affiliations:** Stanford University, Stanford, California, United States

## Abstract

Most research on age stereotyping is based on surveys that ask participants to imagine “an older person” and respond to questions about this hypothetical person. In the present study, we examine whether younger adults’ responses differ when they are asked to imagine hypothetical older people versus presented with an image of a single individual. We also examine how ratings differ when participants evaluate individuals versus generalized representations of older people. Additionally, we examine how these age-based stereotypes are tied to feelings of intergenerational affinity. 288 young participants, aged 18-35 years, were randomly assigned to one of four conditions and asked to rate six positive and six negative traits in response to experimental cues. Experimental cues varied by condition. In two conditions, participants viewed 36 faces presented as either individuals or representatives of their generation. In two other conditions, participants provided ratings about text cues, i.e., an older person or the older person’s generation. Additionally, participants completed a measure of intergenerational affinity. Presentation condition significantly influenced beliefs. Individual presentations elicited less negative beliefs than generalized representations, with individual faces yielding the least negative ratings across all conditions. Conversely, responses to hypothetical representations were less positive than to individual faces. Across conditions, positive perceptions of older people were associated with greater intergenerational affinity. These findings suggest that beliefs about individuals tend to be more positive than beliefs about the category of “older people.” Thus, age integration may more effectively reduce age stereotyping than general discussions about older people.

